# Application of Olfactometry to Assess the Anti-Odor Properties of Filtering Facepiece Respirators Containing Activated Carbon Nonwovens

**DOI:** 10.3390/ijerph18158157

**Published:** 2021-08-01

**Authors:** Małgorzata Okrasa, Justyna Szulc, Agnieszka Brochocka, Beata Gutarowska

**Affiliations:** 1 Department of Personal Protective Equipment, Central Institute for Labor Protection—National Research Institute, Wierzbowa 48, 90-133 Łódź, Poland; agbro@ciop.lodz.pl; 2 Department of Environmental Biotechnology, Lodz University of Technology, 90-924 Łódź, Poland; justyna.szulc@p.lodz.pl (J.S.); beata.gutarowska@p.lodz.pl (B.G.)

**Keywords:** airborne odorous compounds, odor nuisance, filtering respiratory protective devices, olfactometry, workplace environment

## Abstract

Filtering facepiece respirators (FFR) with anti-odor properties are used to reduce odor nuisance occurring both in everyday life and at workplaces. Unfortunately, there are no standardized methods to measure the efficiency of odor reduction of such personal protective devices. This paper aims to determine whether olfactometric-based methods, commonly used in environmental studies, can be employed for this purpose. The proposed procedure is based on the detection of n-butanol by study participants, and it consists of three subsequent stages: (i) defining the individual levels of odor sensitivity of each study participant; (ii) determining THE odor detection level while using FFRs with varying anti-odor properties; and (iii) completing a questionnaire concerning the subjective perceptions of study participants. As a measure of odor reduction efficiency, a coefficient W, defined as a quotient of the degree of odor reduction by the FFR, and the individual odor sensitivity of the subject, was proposed. The experimental results showed the ability of our measure to differentiate the effectiveness of odor reduction of tested FFRs. This indicates that it can be potentially employed as the assessment tool to confirm the effectiveness of such respiratory protective devices as a control measure mitigating the adverse effects of malodors on workers’ health, cognition, and behavior.

## 1. Introduction

Odor pollution has become a significant socio-environmental and public health issue. The sources of malodor include both natural and man-made activities. Those found at workplaces are usually anthropogenic and arise mainly from activities of chemical, petrochemical [1,2], pharmaceutical, food, and plastic processing [3,4,5,6] industries, as well as agriculture [7,8,9,10]. In addition, significant quantities of odorous compounds come from waste processing and storage [11,12,13] and wastewater treatment plants [14,15,16].

Independent of their origin, malodors can elicit negative responses in the human body, cognition, and behavior due to toxicological effects, innate aversion, conditioning or stress responses, and reactions to non-odorous components such as endotoxins [17,18]. Malodors have been shown to cause a range of somatic symptoms, including irritation of the eyes, nose, and throat, headaches, nausea, drowsiness, diarrhea, chest tightness, palpitations, shortness of breath, and sleep disorders [19,20,21]. They have also been shown to aggravate asthma symptoms [20], adversely affect the immune system [22], and cause elevation of stress levels mainly in relation to perceived toxicological effects [23]. In some individuals, malodors may induce self-reported feelings of depression, tiredness, confusion, and even aggression [24,25]. Exposure to intense odors can also affect the performance of complex and short-term memory tasks [26] and modulate reaction time [27,28].

To mitigate the adverse effects of malodor exposure on the health and wellbeing of workers and neighboring household occupants, industrial facilities implement odor control measures [29,30]. The standard hierarchy of controls is usually pursued in those cases, starting from the most effective ones (i.e., elimination or substitution of sources, isolation, and administrative controls) and proceeding to the least effective (i.e., collective protective measures and personal protective equipment). Among the above-mentioned, disposable filtering facepiece respirators (FFRs) with anti-odor properties are the most common for economic reasons and the ease of implementation in an industrial environment [31]. In addition to polymer filtering materials of varying effectiveness, they also contain a layer (or several layers) of activated-carbon-loaded nonwovens, which adsorbs some of the volatile chemical compounds from the breathing air that would otherwise be inhaled.

Only limited research is available on the methods that could be used to assess the odor adsorption efficiency of such FFRs [32,33,34]. They are primarily instrumental and require time-consuming procedures and precise apparatus with very low detection thresholds. Furthermore, due to the complicated relationship between the adsorption capacity of activated carbon and operational conditions of FFRs at the workplace, the results obtained in the laboratory do not give a clear indication of the performance of such devices in practical use [35].

At the same time, odor interactions and deodorization efficiency assessment methods are very well established in environmental studies [36,37,38,39]. Here, besides instrumental methods used to determine the concentrations of individual chemical compounds in air (e.g., chromatography, colorimetry, and sensor-based techniques) [40], olfactometric methods are employed. This category includes dynamic olfactometry, field studies, and odor intensity scaling [4,39].

Our research aimed to determine whether olfactometric methods, generally used for environmental odor assessment, can be employed to determine the odor reduction efficiency of FFRs with anti-odor properties. For this study, we modified one of the methods used to test the olfactory ability of odor inspection staff. In addition, our method included the assessment of practical aspects related to the use of FFRs, such as leak-tightness, comfort, and subjective perception of odor reduction. We used this method to evaluate the effectiveness of three FFRs with different anti-odor properties. The experimental results were then analyzed in terms of the ability to differentiate the effectiveness of odor reduction of these FFRs.

## 2. Materials

### 2.1. Filtering Facepiece Respirators

Three types of filtering facepiece respirators (FFRs) were used for the study: two FFRs with anti-odor properties containing an activated carbon layer and a reference FFR without the activated carbon layer. Anti-odor FFRs were selected to differ significantly in sorption properties (the same amount of activated carbon but with a much different surface area). The design characteristics of the FFRs are shown in Table 1, and their essential protection and functional parameters in Table 2.

### 2.2. Study Group Characteristics

**Figure 1:**
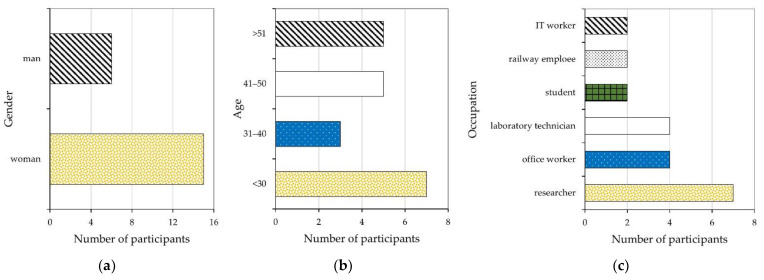
Study group characteristics: (**a**) gender, (**b**) age, and (**c**) occupation.

The study was performed on a group of 21 volunteers. Before the tests, subjects filled out a short questionnaire about gender, age, occupation, and health status. In addition, they were asked not to eat or drink in the half hour directly preceding the planned tests. The characteristics of the study group are shown in Figure 1.

The study group was heterogeneous in age (24–60 years), gender, and occupation. Among the group, only one person was a smoker; two reported to have been unwell in the week before (rhinitis), while one reported symptoms of the common cold.

## 3. Methods

**Figure 2:**
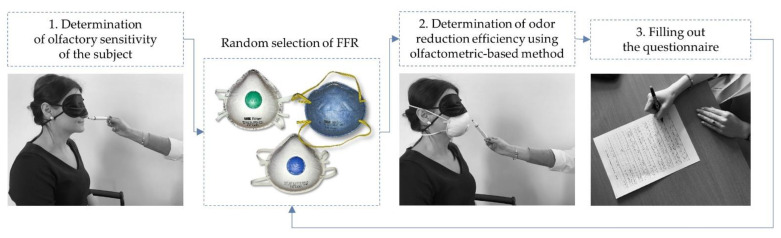
FFR evaluation procedure.

The proposed methodology for assessing odor reduction by using FFRs included three stages, shown schematically in Figure 2.

First, the participants were informed about the aim of the study, and all of the assessment stages were discussed. Next, the participants were instructed on how to put on and check the fit of the FFR. Individual odor sensitivity levels were determined in the first stage of the assessment (see detailed description in Section 3.1). This helped identify the level on the concentration scale at which a test substance could be detected by the participant in a situation in which they did not use an FFR (reference level). Next, the FFR odor reduction efficiency was evaluated, considering each subject’s odor sensitivity level (Section 3.2). The final element of the assessment involved subjects completing a questionnaire regarding factors that may affect odor reduction efficiency by a given FFR and their subjective assessment of the degree of odor reduction when using FFR (Section 3.3).

### 3.1. Determining Individual Odor Sensitivity Levels

The procedure for detecting n-butanol was used to determine participant’s individual odor sensitivity levels [41,42]. It combined two standard procedures. The first, called the method of ascending concentrations, uses 14 pens containing increasing concentrations of the test substance [43]. The second, called the three-alternative forced-choice (3-AFC) [44], requires the participant to indicate which of the three pens generates odors, even if they cannot sense them. The tests were carried out using the Odor Pen Test Kit (St. Croix Sensory, Inc. Stillwater, MN, USA) consisting of 14 instruments (hereafter referred to as pens), containing an increasing concentration of n-butanol, numbered from 15 (lowest concentration) to 2 (highest concentration). In addition, two blank pens filled only with distilled water and an odorless solvent were included in the kit.

Following the warm-up round, the test included two subsequent rounds separated by a 5-min break. In each round, the pens were presented for 3 s at a distance of 1 cm from each of the nostrils of the blindfolded participant in decreasing dilution order. Thus, three pens were presented at each dilution level—two blank ones and one containing n-butanol. The order of pen presentation was determined by one of the three randomly selected results sheets provided along with the test kit [42].

The participants were supposed to remember the corresponding pen number (first, second, third) for which they detected n-butanol odor and indicate whether they could detect the odor to a palpable degree (answer: I am sure/I have detected the pen, marked as D on the results sheet) or made a random choice (the answer: I am not sure/I am guessing, marked as G on the result sheet). The round ended when the subject correctly detected (D) two pens of successive dilutions.

Each round’s individual odor sensitivity level was determined as the number of the first of two consecutive correct detects. The arithmetic mean of the results obtained in rounds 1 and 2 constituted the result.

### 3.2. Assessment of FFRs Odor Reduction Efficiency

The individual odor sensitivity level determination method was modified to determine the odor reduction efficiency of the FFRs. The same measuring tools (Odor Pen Test Kit) and a procedure that combined the ascending concentration method with the 3-AFC method were used [42]. First, the test subject was blindfolded, then the tested FFR was randomly selected (MB 20V, MB 10VC, or 3M 9915) and donned by the participant. Next, the participant was asked to check the fit of the FFR according to the commonly used seal check method [45].

**Figure 3:**
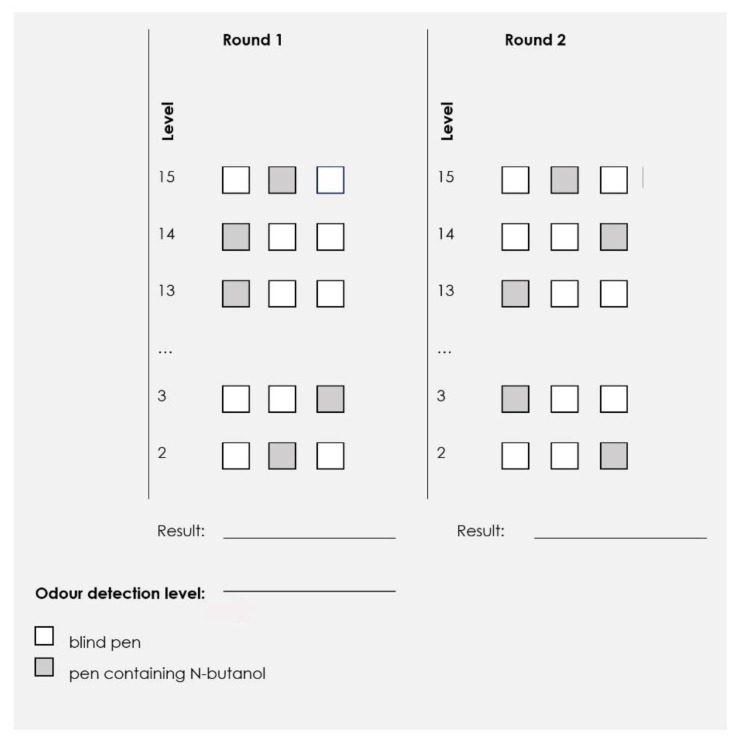
Results sheet for the assessment of the odor reduction efficiency of FFRs.

The test procedure did not require a warm-up round, as the participants were already familiar with the practical aspects of the experiment. Thus, the study was performed in two consecutive rounds, separated by a 5-min break. The pens were presented in triplicates (two blind and one containing n-butanol) according to decreasing dilution order. The order of pen presentation was determined by randomly selecting one of the three specially designed results sheets (e.g., is shown in Figure 3). The reference dilution level from which the test started was one level higher than the individual odor sensitivity level determined beforehand for each participant. The test administrator informed the subjects of the test, beginning by indicating the pen number (e.g., “pen number one”) and instructed them by issuing the command “sniff”. The pen was presented at a distance of 1 cm from the surface of the FFR in an area situated 5 cm below the nose clip. The presentation time was adjusted each time to a period necessary for the participant to take two full inhalations through the nose. Next, the pen was removed from the vicinity of the FFR, and the participant was asked to complete one more breathing cycle to clear the breathing zone (the space situated under the facepiece) of any remnants of the test substance. The participants were supposed to memorize and indicate the pen number at which they detected n-butanol. The answer (sure D and random G) was recorded on the results sheet. Next, pens from successive dilution levels were presented. The rounds ended when the subject correctly detected two pens of successive dilutions.

The test result was determined as the number of the first of two consecutive correct detects. An arithmetic mean of the results obtained in rounds 1 and 2 constituted the result (odor detection level).

The odor reduction degree (ORD) was defined as the difference between individual odor sensitivity levels (ISL) of participants and the odor detection level obtained with the selected FFR (FFRSL). As a measure of odor reduction efficiency (W) of a given FFR, a value defined as follows was calculated:

(1) $$W=\overset{n}{\underset{i}{\sum }}\frac{1}{n}\frac{{\mathrm{ORD}}_{i}}{{\mathrm{ISL}}_{i}}\;\cdot 100\%=\overset{n}{\underset{i}{\sum }}\frac{1}{n}\frac{{\mathrm{ISL}}_{i}-{\mathrm{FFRSL}}_{i}}{{\mathrm{ISL}}_{i}}\;\cdot 100\%,$$

where n is the number of study participants, ISL_i_ is the individual odor sensitivity level of the specified study participant, and FFRSL_i_ is the odor detection level obtained for the specified participant while using FFR.

### 3.3. Questionnaire

The final stage of the evaluation included filling out the questionnaire, which set out what factors affect odor reduction efficiency and how the participants perceive this for a given type of FFR. The questionnaire was composed of four questions regarding the leak-tightness of the facepiece, donning and fitting difficulty, and subjective perceptions of odor reduction (subjective odor reduction efficiency). The answers were ranked using a five-level Linkert’s scale (very high, high, average, low, and very low; if the leak-tightness was assessed as very high, it meant that the panelists considered the fit of the facepiece to their face as very good; if it was assessed as very low, it meant that the fit was considered as very poor) [46]. Additional questions included whether the head harness elastic band snapped or the FFR slipped during the test and whether there was any pungent or unpleasant odor emitted by the FFR itself (evaluation on a yes/no scale).

### 3.4. Statistical Analysis

Statistical analyses were performed using STATISTICA 13.1 software (Statsoft, Tulsa, OK, USA). Descriptive statistics for all variables of interest were calculated. One-way analysis of variance (ANOVA) at the significance level of 0.05 was performed to identify statistical differences between parameters describing odor reduction efficiency of the FFRs. When statistical differences were detected (*p* < 0.05), mean values were compared using Tukey’s post hoc procedure at the significance level of 0.05.

## 4. Results and Discussion

### 4.1. Individual Odor Sensitivities

**Figure 4:**
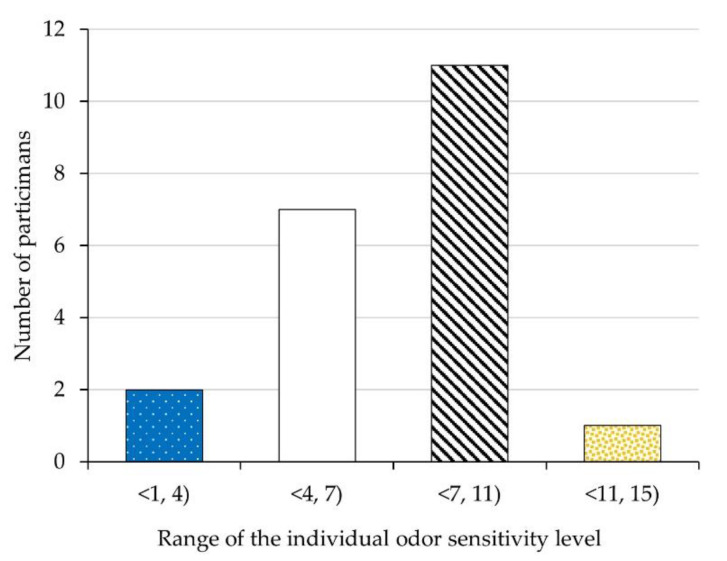
Individual odor sensitivity levels of the study participants.

The analysis of the test results showed that the 7th dilution level constituted the 50th percentile of the studied population, while levels 4 and 11 were the lower and upper 5th percentile, respectively (Figure 4).

The results of published clinical trials on 1036 subjects [47] and the studies of 39 odor inspectors from six Regional Offices of the U.S. Regulation Enforcement Agency [48] show that for all populations studied, the 8th dilution level usually constitutes the 50th percentile of a study population, while levels 3 and 13 are the lower and upper 5th percentiles, respectively. These values constitute normative values for the general population and can indicate performance criteria for odor inspection staff. These are also very close to the values we obtained. Since the score of 0 to 4 is regarded as an indicator of abnormal olfactory function, which in this case can be associated with recent or ongoing viral infection [47], we decided to exclude two participants whose individual levels of olfactory sensitivity fell into the lower 5th percentile of the studied population.

### 4.2. Odor Reduction Efficiency

Table 3 shows the basic statistics describing the odor reduction efficiency of the FFRs based on the developed method.

The highest odor detection levels were obtained for the reference FFR that did not contain the activated carbon layer, while the 3M 9915 FFR had the lowest. Statistically significant differences in odor reduction degree between the reference FFR and FFRs containing activated carbon layers were observed. The lowest values of this parameter were seen for the reference FFR and higher for MB 10VC and 3M 9915 FFRs. Interestingly, the study shows that there is a reduction in odor even with masks that do not contain an activated carbon layer (MB 20V). Part of this reduction can be undoubtedly a result of the test odor provision method (some of the test odor dissipates before it can be aspirated trough the mask), it may also stem from the difference in pressure between the ambient air and the breathing zone, where the so-called ‘dead space’ with limited air exchange occurs. No differences were found between MB 10VC and 3M 9915 FFRs for this parameter (one-way ANOVA, Tukey test, α = 0.05). This means that the degree of odor reduction is not a good measure of actual odor reduction, as it does not differentiate between different types of FFRs. Instead, we found statistically significant differences while comparing the odor reduction efficiencies W for tested FFRs (one-way ANOVA, Tukey test, α = 0.05). For the MB 10VC, it is was higher than the reference by 17%, while for 3M 9915, by 36%. Thus, the results indicate that such a coefficient constitutes a reasonable measure differentiating the efficiency of FFRs with anti-odor properties. They also serve as a premise to continue working in this area to define the criteria for assessing odor reduction efficiency for this personal protective device using olfactometric methods.

### 4.3. Subjective Assessment of Odor Reduction

**Figure 5:**
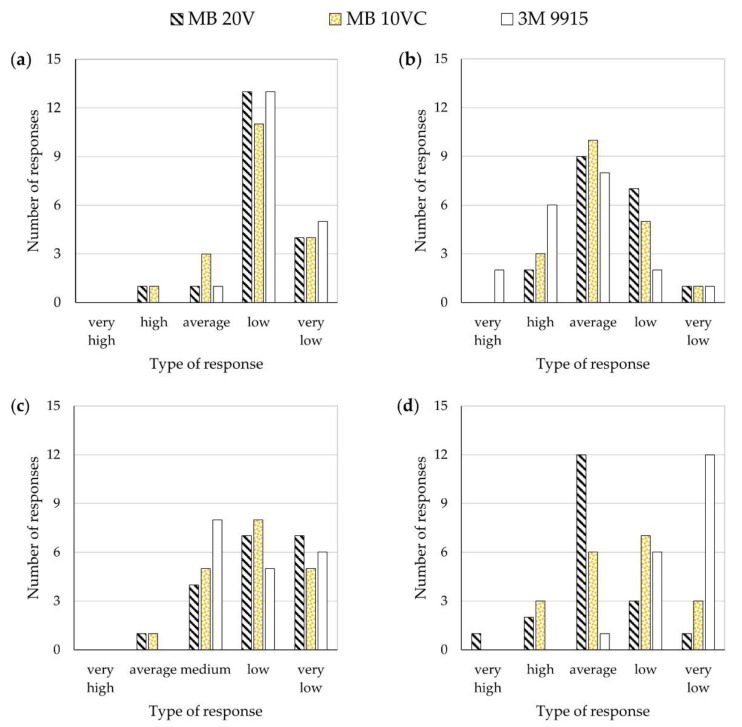
Questionnaire results: (**a**) leak-tightness, (**b**) comfort, (**c**) donning and fitting difficulty level, and (**d**) perception of odor reduction.

The questionnaire results, in which answers were based on a Likert scale, are presented in Figure 5.

There were no instances of head harness elastic band snapping or facepiece sliding. None of the subjects indicated that the reference FFR emits any pungent or unpleasant odors. In contrast, there was one such indication for the MB 10VC FFR and three for the 3M 9915 one. Most study participants pointed to the low leak-tightness of FFRs, which would significantly influence the degree to which such devices limit malodors at a workplace. The comfort of using the FFRs depended on the type of equipment used and the facial features of the participant. The donning and fitting difficulty was assessed as low to average, depending on whether or not the participants were well acquainted with such devices. Considerable individual variability in the subjective assessment of odor reduction of the FFRs was observed. 

The summary of the comparative analysis of the results is shown in Table 4. The following criteria were used for the analysis: the answers “very high” were assigned the grade of 5; “high”—4; “average”—3; “low”—2 and “very low”—1. Next, the grades assigned to individual FFR variants were averaged and analyzed statistically.

Comparative analysis indicated no statistically significant differences in the subjective assessment of the leak-tightness and donning and fitting difficulty between individual FFR types. In terms of the comfort of use, the differences were demonstrated only for the reference variant and 3M 9915 FFR. Regarding the subjective degree of odor reduction, the 3M 9915 FFR was rated the best, while the reference FFR was the worst. There were also statistically significant differences between individual equipment types, consistent with the results obtained based on the olfactometric method.

## 5. Conclusions

Proper control of workplace exposures to malodors can reduce the risk of adverse health effects among employees. In situations where organizational and technical control measures are insufficient or not feasible from an economic point of view, FFRs with anti-odor properties provide an easy option to mitigate the risks. Depending on the region of the world, different legal regulations ensure the effectiveness of FFRs before they are placed on the market. For example, in the United States, respiratory protective devices are certified following the requirements described in Title 42 of the Code of Federal Regulations part 84. At the same time, in the European Union, RPDs should bear Conformitè Europëenne (CE) marking, which generally certifies its compliance with the relevant European standards. Different national requirements are in force in other countries. None of the globally existing normative documents considers methods for determining and approving the ability of a dust mask to capture odorous compounds. Thus, many brands are available for purchase that have not been assessed in terms of odor reduction efficiency; therefore, their effectiveness remains unclear.

This study aimed to verify whether a simple olfactory-based method can be utilized for assessing odor reduction efficiency. The proposed research methodology included three consecutive stages (determining a subject’s odor sensitivity level, evaluating odor reduction efficiency of an FFR, and completing a questionnaire on the subjective perceptions of the participant). The coefficient W of odor reduction efficiency, defined as the quotient of the degree of odor reduction by the FFR and the individual odor sensitivity level of the participant, expressed as a percentage, was proposed as a measure of odor reduction. Based on the analysis of the test results, it was found that this coefficient is a good measure to differentiate the odor reduction efficiency of FFRs with anti-odor properties.

Further work in developing olfactometric-based methods is needed to determine the criteria for assessing the odor reduction efficiency of such types of FFRs. In particular, better means of test odor provision ensuring uniform aspiration conditions while testing panelists with and without FFR (e.g., through a large funnel) should be developed. Moreover, a broader choice of test odors should be considered. In our study, n-butanol was used because it is commonly utilized for odor testing and is an excellent substance to challenge the human olfactory system without unnecessary risk to the test participant. Unfortunately, it can be insufficient to test FFRs ability to remove a large variety of odors occurring at different types of workplaces. Therefore, it would be advisable to use other test substances with different odor characteristics and chemical properties as an alternative to n-butanol (e.g., thiols and terpenes) to better represent the conditions of actual use of this type of FFRs. Additionally, comparing the olfactometric method with functional tests in a natural work environment would also be necessary to validate the method and determine the results’ repeatability and reproducibility.

## Figures and Tables

**Table 1:** The characteristics of FFRs.

Manufacturer/ Type/ Protection Class	Construction	Configuration of Filtering Materials	Characteristics of the Activated Carbon Layer
MB Filter type MB 20V FFP2	Cup-type FFR (cup surface 184 ± 7 cm^2^), equipped with one exhalation valve, adjustable nose clip, two head harness rubber bands, and a facepiece without seals	Spun-bonded nonwoven High efficiency melt-blown nonwoven Spun-bonded nonwoven Calendered needle-punched nonwoven	Not applicable
MB Filter type MB 10VC FFP1		Spun-bonded nonwoven High efficiency melt-blown nonwoven Spun-bonded nonwoven Polymer nonwoven loaded with activated carbon adsorbent material Calendered needle-punched nonwoven	Surface mass: 214 ± 3 g/m^2^ Thickness: 1.9 ± 0.2 mm Carbon content: circa 35% Carbon mass: 1.4 ± 0.1 g Carbon BET surface area: 43 m^2^/g
3M type 9915 FFP1	Cup-type FFR (cup surface 187 ± 1 cm^2^), valveless, equipped with an adjustable nose clip, two head harness textile bands, and a facepiece sealed under the nose clip	Spun-bonded nonwoven High efficiency melt-blown nonwoven Melt-blown nonwoven containing powdered activated carbon adsorbent High efficiency melt-blown nonwoven Needle-punched nonwoven	Surface mass: 216 ± 19 g/m^2^ Thickness: 1.2 ± 0.1 mm Carbon content: circa 35% Carbon mass: 1.4 ± 0.1 g Carbon BET surface area: 651 m^2^/g

**Table 2:** Essential protection and functional parameters of the FFRs.

FFR Type	Parameter	Paraffin Oil Mist Penetration, %	Inhalation Resistance, Pa		Exhalation Resistance, Pa	CO_2_ Content in the Inhaled Air, %
			30 L/min	95 L/min	160 L/min	
MB 20V	N	7	7	7	7	9
	M	0.45 ^a^	46.84 ^a^	151.93 ^a^	177.51 ^a^	0.68 ^a^
	SD	0.22	6.99	19.11	9.22	0.04
	Max	0.85	57.10	173.30	190.10	0.76
	Min	0.23	40.50	132.80	165.50	0.64
MB 10VC	N	10	10	10	10	9
	M	1.56 ^b^	27.60 ^b^	91.94 ^b^	122.72 ^b^	0.77 ^b^
	SD	0.07	1.42	3.98	2.99	0.05
	Max	1.70	29.30	96.60	128.30	0.84
	Min	1.50	24.70	82.90	119.30	0.69
3M 9915	N	10	10	10	10	9
	M	4.27 ^c^	42.08 ^a^	145.52 ^a^	227.04 ^c^	0.79 ^b^
	SD	0.42	3.92	7.28	10.71	0.02
	Max	4.70	47.20	157.50	247.20	0.81
	Min	3.40	34.30	137.10	207.30	0.75

N—number of samples tested, M—mean, S.D.—standard deviation, Max—maximum, Min—minimum, ^a, b, c^—means marked with different letters within the same parameter are statistically different (ANOVA, α = 0.05; Tukey test, α = 0.05).

**Table 3:** The results of the odor reduction efficiency of the FFRs.

FFR Type	Parameter		N	M	SD	Med	Max	Min
MB 20V	Odor detection level	1st round	19	4.16	1.57	5.0	6.0	1.0
		2nd round		4.63	1.67	4.0	8.0	2.0
	Average level of odor detection			4.39	1.36	4.5	6.5	1.5
	Odor reduction degree			2.71 ^a^	1.55	2.5	6.0	0.5
	Odor reduction efficiency W, %			37.21 ^a^	18.24	36.4	70.0	7.1
MB 10VC	Odor detection level	1st round	19	3.00	1.49	3.0	6.0	1.0
		2nd round		3.26	1.45	3.0	5.0	1.0
	Average level of odor detection			3.13	1.41	3.0	5.5	1.0
	Odor reduction degree			3.97 ^ab^	2.04	4.0	8.0	0.5
	Odor reduction efficiency W, %			54.25 ^b^	22.04	57.1	88.9	12.5
3M 9915	Odor detection level	1st round	19	1.63	0.90	1.0	4.0	1.0
		2nd round		1.84	1.07	1.0	4.0	1.0
	Average level of odor detection			1.74	0.93	1.5	4.0	1.0
	Odor reduction degree			5.37 ^b^	2.22	5.0	10.0	1.5
	Odor reduction efficiency W, %			73.24 ^c^	16.92	80.0	90.9	37.5

N—number of samples tested, M—mean, S.D.—standard deviation, Med—median, Max—maximum, Min—minimum, ^a, b, c^—means marked with different letters within the same parameter are statistically different (ANOVA, α = 0.05; Tukey test, α = 0.05).

**Table 4:** Cumulative evaluation of FFRs based on the questionnaire results.

FFR Type	Leak-Tightness	Comfort of Use	The Degree of Donning and Fitting Difficulty	Perception of Odor Reduction
MB 20V	4.1 ^a^ ± 0.7	3.3 ^a^ ± 1.1	1.9 ^a^ ± 0.9	3.1 ^a^ ± 0.8
MB 10VC	4.0 ^a^ ± 0.8	3.2 ^ab^ ± 1.0	2.1 ^a^ ± 0.9	3.5 ^b^ ± 1.0
3M 9915	4.3 ^a^ ± 0.5	2.7 ^b^ ± 1.2	2.1 ^a^ ± 0.9	4.6 ^c^ ± 0.6

^a, b, c^—means marked with different letters within the same parameter are statistically different (ANOVA, α = 0.05; Tukey test, α = 0.05).

## Data Availability

The data presented in this study are available on request from the corresponding author.
